# Daratumumab for CD20 ^−^ CD38 ^+^ Relapsed/Refractory Diffuse Large B‐Cell Lymphoma

**DOI:** 10.1111/jcmm.70875

**Published:** 2025-10-16

**Authors:** ZeTong Hong, ZhaoYang Hong, YaXian Ma, Qing Yin, Min Liu, Wei Huang, KuangGuo Zhou, Dan Li, TongJuan Li, Miao Zheng

**Affiliations:** ^1^ Department of Hematology, Tongji Medical College Tongji Hospital, Huazhong University of Science and Technology Wuhan China; ^2^ Tongji Medical College Huazhong University of Science and Technology Wuhan China

**Keywords:** CAR‐T therapy, CD20, CD38, chemotherapy, immunotherapy

## Abstract

Patients with R/R DLBCL (relapsed/refractory diffuse large B‐cell lymphoma) treated with rituximab‐based regimens often have a reduction in the expression level of CD20. Replacement of target proteins for new targeted chemotherapy has become a popular direction for the treatment of R/R DLBCL. To investigate whether Daratumumab can be an effective alternative to targeted agents as monotherapy or combination chemotherapy in patients with CD20‐CD38+ R/R DLBCL who have failed to respond to CD20 monoclonal antibody treatment and its adjuvant effect on subsequent CAR‐T (chimeric antigen receptor T‐cell immunotherapy). A total of four CD20‐CD38+ R/R DLBCL patients treated with multiple lines of Daratumumab‐based combination chemotherapy were retrospectively collected. For eligible patients, CAR‐T therapy was used afterwards. Also, the authors successfully constructed allografted tumour models in mice. Relevant evaluation showed that four patients who received Daratumumab combination chemotherapy had varying degrees of remission after treatment, including 2 CR, 1 PR and 1 SD. The 1‐year OS rate was 75%, the 1‐year PFS rate was 50% and mOS was 12 months. Adverse effects were moderate and reversible, and no treatment‐related deaths occurred. Daratumumab therapy led to a successful transition to CAR‐T in two patients. Among them, grade 1 CRS occurred. These cases demonstrated that Daratumumab combination therapy has a good application prospect in the treatment of CD20‐CD38+ R/R DLBCL and is helpful for the bridge of CAR‐T therapy.

**Abbreviations**
AE: adverse event
CAR‐T: chimeric antigen receptor T‐cell
CBR: clinical benefit rate
CNS: central nervous system
CR: complete remission
HSCT: haematopoietic stem cell transplantation
ORR: overall response rate
OS: overall survival
PD: progressive disease
PFS: progression‐free survival
PR: partial remission
R/R DLBCL: relapsed/refractory diffuse large B‐cell lymphoma
SD: stable disease
TEAE: treatment‐emergent adverse event

## 1 Introduction

Diffuse large B‐cell lymphoma is a highly aggressive non‐Hodgkin lymphoma. At present, the standard first‐line treatment is Rituximab targeting CD20. About 30%–40% of patients will progress to R/R DLBCL, among which 60% have a downregulation in CD20 with insensitivity to rituximab treatment. Salvage treatment includes replacement of chemotherapy (DA‐EPOACH, etc.) or HSCT (Haematopoietic Stem‐Cell Transplantation). The prognosis of patients is still poor: the 3‐year OS rate is 38%–50%, the 3‐year PFS rate is 29%–37%. Therefore, for R/R DLBCL with downregulation of CD20, the replacement of therapeutic targets has become a major direction of clinical research. At present, Polatuzumab targeting CD79b, Tafasitamab targeting CD19 [1] and Daratumumab targeting CD38 have entered clinical trials [2].

CD38 is a glycoprotein located on the cell membrane. It has been reported that the expression of CD38 in most malignant blood tumour cells is much higher than in normal cells, including WM, ALL, AML and various types of B‐cell lymphomas [2, 3, 4, 5, 6, 7]. Importantly, CD38 is not expressed by multipotent haematopoietic stem cells, which are essential for long‐term bone marrow recovery [8]. These make CD38 an attractiv.e therapeutic target for R/R DLBCL.

Daratumumab is a CD38 monoclonal antibody approved as regimens for patients with MM who have received at least three previous therapies [9]. Daratumumab induced tumour cell apoptosis through a variety of mechanisms, including complement‐dependent cytotoxicity, antibody‐dependent cell‐mediated cytotoxicity, antibody‐dependent cell phagocytosis and Fcγ receptor‐mediated apoptosis induced by the direct cytotoxicity [10], directly killing tumour cells by activating complement proteins, NK cells and macrophages, as well as by cross‐linking [11]. Daratumumab also abolished the highly immunosuppressive subsets of CD38^+^ Tregs, CD38^+^ MDSC and CD38 regulatory B cells (B‐reg) [12].

Many studies have shown that Daratumumab can effectively inhibit the growth of B cell tumours. CD38‐CAR‐T cells could significantly inhibit the growth of CD38‐overexpressing MCL cells in an in vitro model and transplanted tumour cells in mice [13]. Another preclinical experiment [14] showed that injection of Daratumumab into an in vitro model of DLBCL tumours resulted in significant tumour growth control. Therefore, Daratumumab may have a positive therapeutic impact on CD20^−^ R/R DLBCL. In addition, Daratumumab depleted the highly immunosuppressive subset of CD38^+^ Tregs, suggesting that Daratumumab may facilitate the proliferation of CAR‐T cells in vivo and play an auxiliary role in CAR‐T treatment.

However, the clinical data on Daratumumab therapy in patients with R/R DLBCL remain limited. Therefore, we evaluated the safety and efficacy of Daratumumab in four patients with R/R CD20^−^CD38^+^DLBCL, to explore the new treatment method for the disease.

## 2 Material and Methods

We collected data from patients treated with Daratumumab for R/R CD20^−^CD38^+^ DLBCL admitted to Wuhan Tongji Hospital between July 2021 and December 2023. They had been treated with at least three lines of chemotherapy (Details of pretime regimens are provided in Appendix C).

The efficacy and adverse reactions of the patients were observed (based on 2014 Lugano standard). Some patients received Daratumumab combined with CAR‐T therapy. The efficacy in these patients was also observed to evaluate the auxiliary effect of Daratumumab on CAR‐T therapy.

Four patients were enrolled in this study; baseline characteristics are shown in Table 1. Patients had been treated with multiple lines of chemotherapy, including rituximab‐based regimens, HSCT and multiple targets for CAR‐T treatment (CD19, CD22, CD79b, CD20). After relapse, the results of flow cytometry showed that the tumour cells were down‐regulated in the expression of targets such as CD20, and CD38 was strongly positive.

**TABLE 1:** Baseline characteristics of the patients.

Baseline characteristics	
Characteristic	
Number	4
Gender	
Male	1 (25%)
Female	3 (75%)
Age	
Median	50.5
< 60 no. (%)	3 (75%)
≥ 60 years no. (%)	1 (25%)
Ann Arbor stage	
I–II	0 (0%)
III–IV	4 (100%)
IPI	
0–1	0 (0%)
2–4	4 (100%)
Prior therapies	
Median (range) lines	4
Preline therapies > 3	4 (100%)
Involvement outside of lymphnodes	
No	0 (0%)
Yes	4 (100%)
Pretreatment disease burden	
Bone marrow involvement	4 (100%)
Double hit	1 (25%)
HSCT pretherapy	1 (25%)

No.	Age	Gender	Subtype	Ann Arbor	IPI	Double hit	
1	46	female	non‐GCB	IVa	2	BCL‐2	C‐Myc
2	55	female	GCB	IVa	3	\	
3	68	female	GCB	IIIa	3	\	
4	46	male	GCB	IVb	4	\	

All patients underwent immunohistochemical analysis of tumour cells before the treatment; details are provided in Appendix A. Results showed that all patients had loss or mutation of CD20 and other targets before medication, which may not be effective for CD20‐containing monoclonal antibody. Although CD19 and CD22 are weakly positive in some patients, their expression rate is much lower than that of the CD38 antigen on the surface of the sample tumour. Therefore, CD38 monoclonal antibody is a good option.

Patients 1 and 2 were applied to Daratumumab combination chemotherapy and CAR‐T treatment; patients 3 and 4 were applied to Daratumumab combination chemotherapy only (without CAR‐T treatment). Details of treatment regimens are provided in Table 2.

**TABLE 2:** Immunohistochemical target assessment and treatment.

No.	Targets evaluation				Treatment					
	CD20	CD19	CD38	CD22	Dara cycles	Combination drug	CART therapy	CD19 (*10^6/kg)	CD22 (*10^6/kg)	CRS level
No. 1	−	+	+	+	3	Orelabrutinib, Selinexor	Yes	2.5	0.5	1
No. 2	−	+	+	+	4	Venetoclax, Gemox	Yes	0.5	/	1
No. 3	−	+	+	+	3	Orelabrutinib	No	/	/	/
No. 4	−	+	+	−	5	Cytarabine, ICE, DA‐EPOCH	No	/	/	/

All patients received Daratumumab 16 mg/kg by intravenous infusion weekly, with Dexamethasone 15 mg 1 h before administration, promethazine 25 mg and loxoprofen sodium tablets 30 mg 30 min before administration.

Follow‐up was conducted until January 2024. Due to the small sample size, we directly calculated 1‐year PFS (progression‐free survival, the time from the start of treatment to tumour progression or last follow‐up), 1‐year OS (overall survival, the time from the start of treatment to the patient's death or last follow‐up) and mOS (median overall survival) according to patients' data.

## 3 Results

### 3.1 Efficacy

**FIGURE 1:**
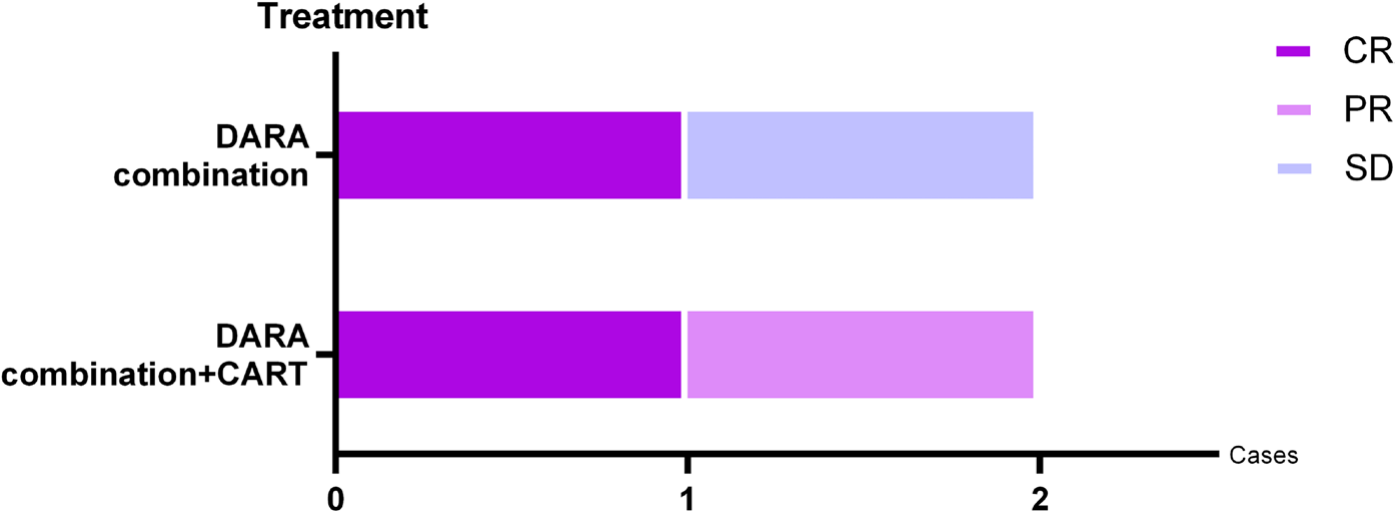
All four patients achieved varying degrees of remission (CR, *n* = 2; PR, *n* = 1; SD, *n* = 1), with an ORR rate of 75% and a CBR rate of 100%.

Specific efficacy is shown in Figure 1. All four patients achieved varying degrees of remission (CR, *n* = 2; PR, *n* = 1; SD, *n* = 1), with an ORR (overall response rate) of 75% and a CBR (clinical benefit rate) of 100%. The 1‐year OS rate was 75%, the 1‐year PFS rate was 50% and mOS was 12 months.

In patients 1–2 (treated with Daratumumab combination chemotherapy and CAR‐T treatment), Daratumumab resulted in disease stabilisation and successful transition to CAR‐T, with tumour burden release after the full treatment (CR, *n* = 1; PR, *n* = 1).

In patient 3–4 (treated with Daratumumab combination chemotherapy only), Daratumumab achieved significant tumour control, 2 patients had clinical benefits (CR, *n* = 1; SD, *n* = 1).

### 3.2 TEAEs

About the TEAEs, no patients died during treatment. During the treatment in this study, patients experienced the following adverse effects: Anaemia (4/4), neutropenia (4/4), thrombocytopenia (4/4), fever (1/4), infusion‐related reactions (1/4) and upper respiratory tract infection (1/4). Details are provided in Appendix B.

**FIGURE 2:**
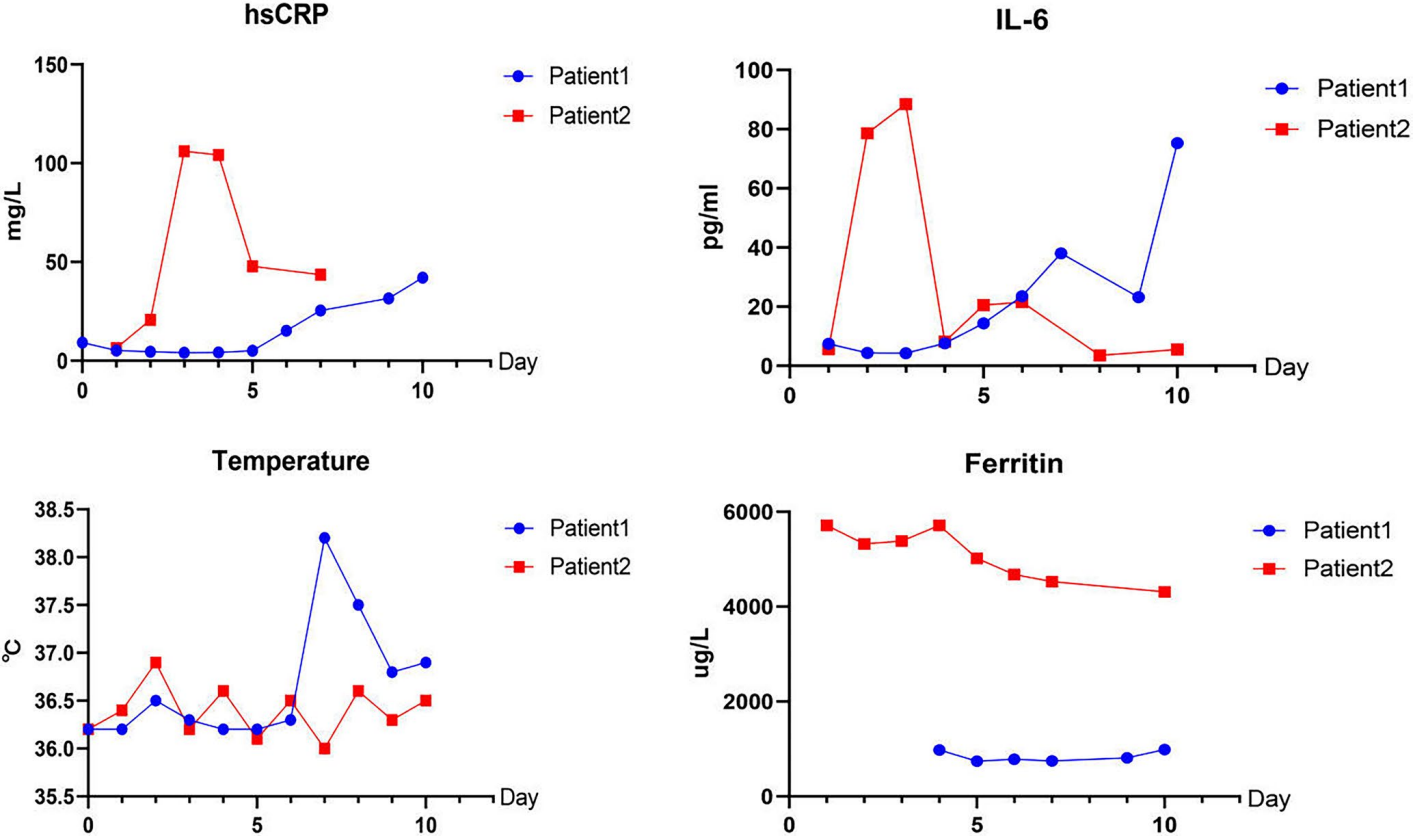
Patients 1 and 2 developed grade 1 CRS during the subsequent bridging CAR‐T therapy.

Two patients were treated with Daratumumab‐containing regimens, and all successfully transitioned to CAR‐T with grade 1 CRS. Detailed data related to CRS in these patients were provided in Figure 2.

### 3.3 Additional Animal Experiment

In addition, for the case (Patient 2) involved in this paper, authors Huang Wei, Zhou Kuangguo et al. conducted additional animal experiments [14].

Researchers purchased DLBCL cells with p53 mutations and high expression of CD38 and injected them into 5‐week‐old female NOD‐SCID mice, successfully constructing allografted tumour models in mice. The mice were divided at random into five groups: vehicle group; Daratumumab group, venetoclax group, Daratumumab combined with venetoclax group; and Daratumumab and venetoclax combined with other chemotherapy group. Different regimens of chemotherapy were then applied to each group, and tumour size, tumour weight and mouse body weight were monitored carefully throughout the experiments.

Results came out afterwards. The mice in the Daratumumab combined with venetoclax group had a strong inhibitory effect on tumour growth. In contrast, Daratumumab and venetoclax alone did not significantly inhibit tumour growth.

## 4 Discussion

### 4.1 Efficacy

This is the first case series report of the efficacy of Daratumumab‐contained regimens in patients with R/R DLBCL. Based on many relevant experiments worldwide, Daratumumab has a certain anti‐tumour effect on R/R DLBCL. In this study, the core goal of salvage therapy after relapse is to use new targets or to help patients transition to the subsequent CAR‐T therapy. From this point of view, our findings showed that Daratumumab was successful in accomplishing this goal, helping patients relieve tumour burden and successfully leading to the CAR‐T treatment.

A number of previous clinical trials have verified the positive effect of this therapy. In the experiments done by Vidal‐Crespo A. et al. [15], researchers injected the DLBCL tumour model constructed in vitro with Daratumumab combined with R‐CHOP therapy. Later, the tumour in the experimental group had a relatively significant shrinkage compared with the control group injected only with R‐CHOP. Such experiments indicated that Daratumumab combined with other cytotoxic drugs has a more significant anti‐tumour effect than the traditional R‐CHOP regimen in R/R NHL.

In this study, all patients treated with Daratumumab showed clinical benefit at the subsequent evaluation, indicating that Daratumumab combination chemotherapy has a considerable application prospect in the treatment of R/R DLBCL. The different therapeutic efficacy of each patient may be related to the different basic conditions and the different combination chemotherapy regimens used.

There had been a phase II clinical trial of Daratumumab monotherapy in R/R NHL, using Daratumumab monotherapy to treat FL, MCL and DLBCL. The study met the predefined futility criteria. The possible reason is the difference between MM and NHL's tumour microenvironment. In addition, it may be attributed to the higher and more even CD38 expression rate on MM cells [2], resulting in the less significant effect of Daratumumab monotherapy in NHL than in MM. While using Daratumumab combination therapy, Daratumumab serves as a monoclonal antibody rather than serves as an ADC, which is the reason Daratumumab combination therapy had better efficacy than monotherapy.

Daratumumab also has a certain adjuvant effect on CAR‐T treatment. Daratumumab‐containing regimens can be used to bridge CAR‐T without compromising CAR‐T cell expansion or efficacy (Figure 2). In this case, all two patients successfully transitioned to CAR‐T therapy. Patient 1 and Patient 2 had a fairly positive response to subsequent CAR‐T therapy, and both patients smoothly tolerated CAR‐T therapy and had tumour remission. Relevant in vitro experiments have provided strong evidence for this. Jakub Krejcik et al. noted that CD38‐expressing immunosuppressive regulatory T and B cells and myeloid‐derived suppressor cells were sensitive to Daratumumab treatment; also, cytotoxic T‐cell number, activation and clonality increased after Daratumumab treatment in heavily pretreated relapsed and refractory MM. They described previously unknown immunomodulatory effects of Daratumumab through the reduction of CD38+ immunosuppressive cellular populations and concomitant induction of helper and cytotoxic T‐cell expansion, production of IFN‐γ in response to viral peptides and increased TCR clonality, indicating an improved adaptive immune response [12]. From this point of view, Daratumumab‐containing therapy may be complementary to CAR‐T therapy.

The small sample size is one of the shortcomings of this experiment. Additionally, due to the varying initial treatment plans for the patients, the selected combined chemotherapy drugs were not uniform, which is also a limitation of this study. In the future, we may verify our conclusions through prospective studies with larger sample sizes and more systematic and standardised approaches.

### 4.2 TEAEs

Safety is another advantage of the Daratumumab chemotherapy. The Daratumumab‐containing regimen showed a good safety profile in all patients; no patients died during the treatment.

Daratumumab has been shown to be safe in previous studies of MM, with few AEs associated with severe pancytopenia. In a phase 1/2 trial of Daratumumab, Lenalidomide and Dexamethasone in MM, no dose‐limiting toxicity was observed at the dose of 16 mg/Kg, and in subsequent treatments, Neutropenia was the most frequently reported (78.1%) grade 3/4 TEAE and was considered to be lenalidomide‐related [16]. It is noted that the patients have received multiple lines of chemotherapy previously; their basic state is extremely poor, and Daratumumab is used in combination with other chemotherapy drugs, which causes different AEs from Daratumumab monotherapy, all of which need to be comprehensively considered.

In a study of Daratumumab monotherapy in R/R NHL, the most common grade 3/4 AEs were anaemia, neutropenia and thrombocytopenia [1].

No specific AEs were observed in this study, and the AEs that occurred were easy to control, which led us to believe that the relevant treatment regimen was safe to a certain extent.

## 5 Conclusion

Above all, we can say that Daratumumab combination chemotherapy had a positive effect on four patients with R/R DLBCL and helped two patients to successfully bridge CAR‐T therapy. Overall, these cases demonstrated a good application prospect of Daratumumab in the treatment of CD20^−^CD38^+^ R/R DLBCL and helped bridge CAR‐T therapy.

## Author Contributions

**ZeTong Hong:** conceptualization (lead), data curation (lead), formal analysis (equal), funding acquisition (equal), investigation (equal), methodology (equal), project administration (equal), resources (equal), software (equal), supervision (equal), validation (equal), visualization (equal), writing – original draft (lead), writing – review and editing (lead). **ZhaoYang Hong:** conceptualization (lead), data curation (lead), formal analysis (equal), funding acquisition (equal), investigation (equal), methodology (equal), project administration (equal), resources (equal), software (equal), supervision (equal), validation (equal), visualization (equal), writing – original draft (lead), writing – review and editing (lead). **YaXian Ma:** formal analysis (equal), funding acquisition (equal), investigation (equal), methodology (equal), project administration (equal), resources (equal), software (equal). **Qing Yin:** formal analysis (equal), investigation (equal), methodology (equal), project administration (equal). **Min Liu:** formal analysis (equal), resources (equal), software (equal), supervision (equal). **Wei Huang:** supervision (equal), validation (equal), visualization (equal). **KuangGuo Zhou:** investigation (equal), resources (equal), software (equal), supervision (equal), validation (equal), visualization (equal). **Dan Li:** formal analysis (equal), resources (equal), software (equal), supervision (equal). **TongJuan Li:** conceptualization (equal), data curation (equal), formal analysis (equal), funding acquisition (equal), investigation (equal), methodology (equal), project administration (equal), resources (equal), software (equal), supervision (equal), validation (equal), visualization (equal), writing – original draft (equal), writing – review and editing (equal). **Miao Zheng:** conceptualization (equal), data curation (equal), formal analysis (equal), funding acquisition (equal), investigation (equal), methodology (equal), project administration (equal), resources (equal), software (equal), supervision (equal), validation (equal), visualization (equal), writing – original draft (equal), writing – review and editing (equal).

## Disclosure

The authors declare that they have no known competing financial interests or personal relationships that could have appeared to influence the work reported in this paper.

## Ethics Statement

The study was approved by the institutional review board of Tongji Hospital, Tongji Medical College, Huazhong University of Science and Technology. All procedures followed were in accordance with the ethical standards of the responsible committee on human experimentation (institutional and national) and with the Helsinki Declaration.

## Consent

Informed consent was obtained from all patients included in the study.

## Conflicts of Interest

The authors declare no conflicts of interest.

## Data Availability

Data available on request due to privacy/ethical restrictions.

## Appendix A Immunohistochemical Results

**Table:**

Patients	Targets evaluation					Residual targets	Therapeutic schedule
	CD20	CD19	CD79b	CD38	CD22		
Patient 1	(−) MFI = 457	(+) MFI = 2202	(+) MFI = 2202	Partially (+)	(+) MFI = 1850	CD19 CD22 CD38	DARA+CD19 CD22CART
Patient 2	(−)	(+)	(+)	(+)	(+)	CD19 CD22 CD38	DARA+CD19CART
Patient 3	(−) MFI = 140	(+) (CART*2) MFI = 2091	(+) (CART*2) MFI = 3654	(+) MFI = 7887	(+) (CART*2) MFI = 1608	CD38	DARA
Patient 4	(−)	(+) (CART*1)MFI = 10,899	MFI = 1826	(+) MFI = 6727	MFI = 875	CD38	DARA
Interpretation	Traditional Scheme, all (−)	McAbs not been used clinically (in mainland China)	At the time of treatment of patients in this study, guidelines do not yet recommend this drug (in mainland China)	Dara, (+) OrPartially (+)	The use of related McAbs is less	Almost only CD38	DARA (+CART)

## Appendix B Safety Analysis

**Table:**

Adverse reactions	No.			
	No. 1	No. 2	No. 3	No. 4
Transfusion related reaction			III	
Anemia	II	IV	I	IV
Thrombocytoprnia	IV	IV	I	IV
Neutrophilic granuloctopenia	III	IV	I	IV
Nausea				
Cough				
Fever				II
URI			I	

## Appendix C Pretime Regimens

**Table:**

No.	Pretime regimens	HSCT	CART
1	R‐CEOP90*6	Once	\
	BEAM		
	Obinutuzumab+selinexor+Orelabrutinib		
	Ifosfamide+Etoposide+Obinutuzumab		
2	R‐DA‐EPOCH	\	\
	Rituximab+Lenalidomide+Zanubrutinib		
	R‐DHAP		
	PD‐1+R‐DHAP		
3	R‐CHOP*6	\	Twice
	Chidamide+DICE*2		
	Cytarabine+Methotrexate+Dexamethasone		
	PD1*10		
	Chidamide		
4	R‐CHOP	\	Once
	HD‐MTX*5		
	R‐EPOCH*4		
	Rituximab		
	Chidamide		
	PD‐1*4		
	Chidamide+PD‐1*16		
	Lenalidomide		
	BFM‐90		
	R‐Hype‐CVAD‐B		
	ICE+Chidamide+Zanubrutinib		
